# The role of inflammasome in chronic viral hepatitis

**DOI:** 10.3389/fcimb.2024.1382029

**Published:** 2024-05-16

**Authors:** Pin Wan, Ge Yang, Qi Cheng, Xuelong Zhang, Zhaoyang Yue, Moran Li, Chunlin Liu, Qian Yi, Yaling Jia, Jinbiao Liu, Xiwen Xing, Binlian Sun, Yongkui Li

**Affiliations:** ^1^ Hubei Key Laboratory of Cognitive and Affective Disorders, Institute of Biomedical Sciences, School of Medicine, Jianghan University, Wuhan, China; ^2^ Foshan Institute of Medical Microbiology, Foshan, China; ^3^ Wuhan Jinyintan Hospital, Tongji Medical College of Huazhong University of Science and Technology, Wuhan, China; ^4^ Institute of Medical Microbiology, Department of Immunology and Microbiology, College of Life Science and Technology, Jinan University, Guangzhou, China; ^5^ Laboratory of Viral Pathogenesis & Infection Prevention and Control (Jinan University), Ministry of Education, Guangzhou, China; ^6^ National “111” Center for Cellular Regulation and Molecular Pharmaceutics, Key Laboratory of Fermentation Engineering (Ministry of Education), Hubei Provincial Cooperative Innovation Center of Industrial Fermentation, Hubei Key Laboratory of Industrial Microbiology, Sino-German Biomedical Center, Hubei University of Technology, Wuhan, China; ^7^ Department of Biotechnology, College of Life Science and Technology, Jinan University, Guangzhou, China

**Keywords:** viral hepatitis, NLRP3, inflammasome, IL-1β, IL-18, pyroptosis

## Abstract

Infections of hepatotropic viruses cause a wide array of liver diseases including acute hepatitis, chronic hepatitis and the consequently developed cirrhosis and hepatocellular carcinoma (HCC). Among the five classical hepatotropic viruses, hepatitis B virus (HBV) and hepatitis C virus (HCV) usually infect human persistently and cause chronic hepatitis, leading to major troubles to humanity. Previous studies have revealed that several types of inflammasomes are involved in the infections of HBV and HCV. Here, we summarize the current knowledge about their roles in hepatitis B and C. NLRP3 inflammasome can be activated and regulated by HBV and HCV. It is found to exert antiviral function or mediates inflammatory response in viral infections depending on different experimental models. Besides NLRP3 inflammasome, IFI16 and AIM2 inflammasomes participate in the pathological process of hepatitis B, and NALP3 inflammasome may sense HCV infection in hepatocytes. The inflammasomes affect the pathological process of viral hepatitis through its downstream secretion of inflammatory cytokines interleukin-1β (IL-1β) and IL-18 or induction of pyroptosis resulting from cleaved gasdermin D (GSDMD). However, the roles of inflammasomes in different stages of viral infection remains mainly unclear. More proper experimental models of viral hepatitis should be developed for specific studies in future, so that we can understand more about the complexity of inflammasome regulation and multifunction of inflammasomes and their downstream effectors during HBV and HCV infections.

## 1 Introduction

Viral hepatitis causes significant damage to human health and life safety. There are five main hepatotropic viruses inducing hepatitis, and they are named hepatitis A, B, C, D and E viruses (abbreviated as HAV, HBV, HCV, HDV and HEV respectively). They cause various forms of hepatitis and live-associated diseases, and have frequently emerged as focal outbreaks or initiated epidemic spread. HAV infection usually leads to acute hepatitis, and annually plague 1.4 to 1.5 million people worldwide (Abutaleb and Kottilil, 2020). HBV infects 350 million individuals globally every year (Robinson et al., 2023), and about 5% of the infected people are subsequently plagued by persistent infection and chronic hepatitis B. The chronic HBV infection leads to high risk of developing fatal liver disease including liver failure, cirrhosis and hepatocellular carcinoma (HCC) (Collaborators GBDHB, 2022). HCV infects about 2–3% of the population worldwide, most of which suffer from chronic hepatitis and serious live diseases. Hepatitis C resulted in 350,000 to 400,000 deaths annually (Hanafiah et al., 2013), before the effective anti-HCV drug Sofosbuvir & Velpatasvir was developed and applied (Feld et al., 2015). HCV can be transmitted through transfusion, organ transplantation, intravenous injection and sexual contact (Vilibic-Cavlek et al., 2015). The infection and replication of HDV relies on assistance of other virus, and HDV usually co-infect the HBV carries at a very low rate. HDV is a member of the Deltaviridae family, genus Deltavirus. Despite high rates of HBV infection, there are very low rates of HDV co-infection in other Asian countries, including China. However, HDV is considered to play a promoting role in the pathological process of HBV-associated cirrhosis and HCC in co-infection (Stockdale et al., 2020). HEV includes 8 known genotypes, among which Genotype 1 and 2 are known to infect humans endemically in developing countries (Nimgaonkar et al., 2018), and Genotypes 3 and 4 are known to infect multiple species (Meng, 2016). Among the five types of hepatitis viruses, the infections of HBV and HCV are primarily responsible for chronic hepatitis and long-term healthy damage to human, so we mainly discuss hepatitis B and C here.

Pattern recognition receptors (PRRs), which function as a critical part of innate immune system and recognize pathogen components (Chou et al., 2023; Dubyak et al., 2023). Inflammasomes act as an important type of PRRs in response to pathogen stimulation or cell stress signals (Venuprasad and Theiss, 2021). Inflammasomes are a kind of cytoplasmic multiprotein complexes which typically composed of a sensor molecule (can be NOD-like receptors (NLRs), absent in melanoma 2 (AIM2) or pyrin), an adaptor protein ASC, and an effector protein pro-Caspase-1 (Keestra-Gounder and Nagao, 2023). When inflammasomes are stimulated by pathogen component of cell stress signals, the complexes are assembled, cleaving pro-Caspase-1 into active Caspase-1.Then, the active Caspase-1 can cleave pro-interleukin-1β (pro-IL-1β), pro-interleukin-18 (pro-IL-18) or cytosolic protein gasdermin D (GSDMD) into mature forms IL-1β, IL-18 or N-GSDMD respectively (Xia et al., 2020). The N-GSDMD forms pores on the plasma membrane to induce pyroptosis, which promotes the release of mature IL-1β and IL-18 into the extracellular space (Xia et al., 2020; Chauhan et al., 2022). These two proinflammatory cytokines and pyroptosis may drive the progression of hepatitis B and C if the inflammasome are activated during the infection of hepatitis viruses.

HBV and HCV are majorly responsible for the hepatitis induction (Dadmanesh et al., 2014). Viral infections not only stimulate innate immune responses, but also arouse acute or chronic inflammation in human liver. Chronic hepatitis has been considered as an important driving etiology for liver cirrhosis and HCC in HBV and HCV positive individuals. It has been demonstrated that several types of inflammasomes participate in the pathogenesis of chronic hepatitis, liver cirrhosis and HCC. Here, we summarize current studies that may reveal the role of inflammasome in viral hepatitis and the related liver diseases, point out the inadequacies of the previous researches, and provide prospects for this field.

## 2 NLRP3 inflammasome was closely involved in hepatitis B infection

**Figure 1:**
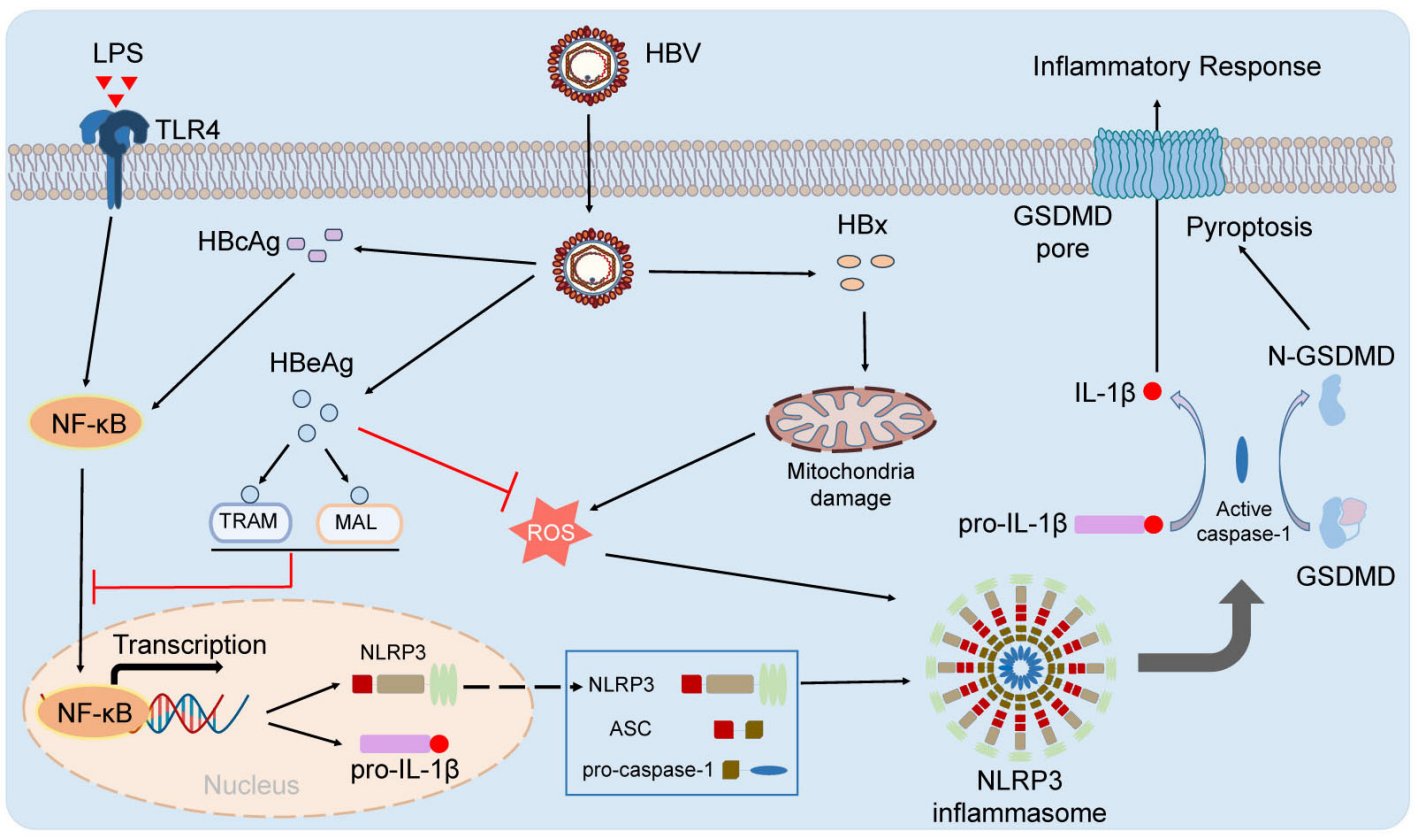
The activation and regulation of NLRP3 inflammasome in HBV infection. HBx and HBcAg triggers the activation of NLRP3 inflammasome, and HBeAg acts as a suppressor of NLRP3 inflammasome through inhibiting its expression and its activation.

HBV infection could induce acute and chronic inflammatory responses in the liver. The acute inflammatory responses were responsible for viral clearances and the chronic inflammatory responses also was helpful for disease pathogenesis (Robinson et al., 2016). HBV has developed strategies to escape immune responses. The NLRP3 inflammasome have reportedly been an important role in inhibiting HBV infection *in vivo* (Watashi et al., 2013). It has been reported that NLRP3 inflammasome was activated in peripheral blood mononuclear cells (PBMCs) from patients infected with acute hepatitis B by quantitative real-time PCR (qRT-PCR) and enzyme-linked immunosorbent assay (ELISA) (Chen et al., 2018). It has been suggested that NLRP3 inflammasome was closely involved in antiviral defense and the pro-inflammatory cytokine IL-1β suppressed HBV infection *in vivo* (Yu et al., 2017). It has also been shown that HBV infection could regulate NLRP3 inflammasome activation and escape host innate immune responses (Yu et al., 2017). Several HBV proteins including the viral polymerase (HBp), x protein (HBx), s antigen (HBs) and e antigen (HBe), could suppress innate immune signaling pathways, leading to viral persistence and immunosuppression (Bertoletti and Ferrari, 2013). Kupffer cells (the liver resident macrophages), but not hepatocytes, could produce significantly amounts of pro-IL-1β and express may kinds of the NLRs (Nod-like receptors), including NLRP3 (Szabo and Csak, 2012). HBV could not activate NLRP3 inflammasome but inhibits LPS-induced the activation of NLRP3 inflammasome in the HBV-persistent mice (Yu et al., 2017). HBeAg of HBV could suppress LPS-induced activation of NLRP3 inflammasome and IL-1β secretion by inhibiting the NF-kB signaling pathway and decreasing the ROS production (Yu et al., 2017). HBcAg also promoted LPS-induced NLRP3 inflammasome activation and IL-1β secretion in the HepG2 cells by regulating NF-kB phosphorylation (Ding et al., 2019). Hepatitis B virus X protein (HBx) is a critical factor for HBV-induced hepatitis (Xie et al., 2020). HBx could activate activation of NLRP3 inflammasome activation and induce hepatocellular pyroptosis by induced mitochondrial damage and production of mitochondrial reactive oxygen species in hydrogen peroxide-stimulated HL7702 cells (Xie et al., 2020). Overall, the detailed regulation of NLRP3 inflammasome in HBV infection appears every complex. For example, HBx and HBcAg triggers the activation of NLRP3 inflammasome, and HBeAg acts as a suppressor of NLRP3 inflammasome through inhibiting its expression and its activation (**Figure 1**). The regulation and the role of NLRP3 inflammasome in Hepatitis B need to be further studied under physiological conditions.

## 3 NLRP3 inflammasome was closely involved in hepatitis C infection

**Figure 2:**
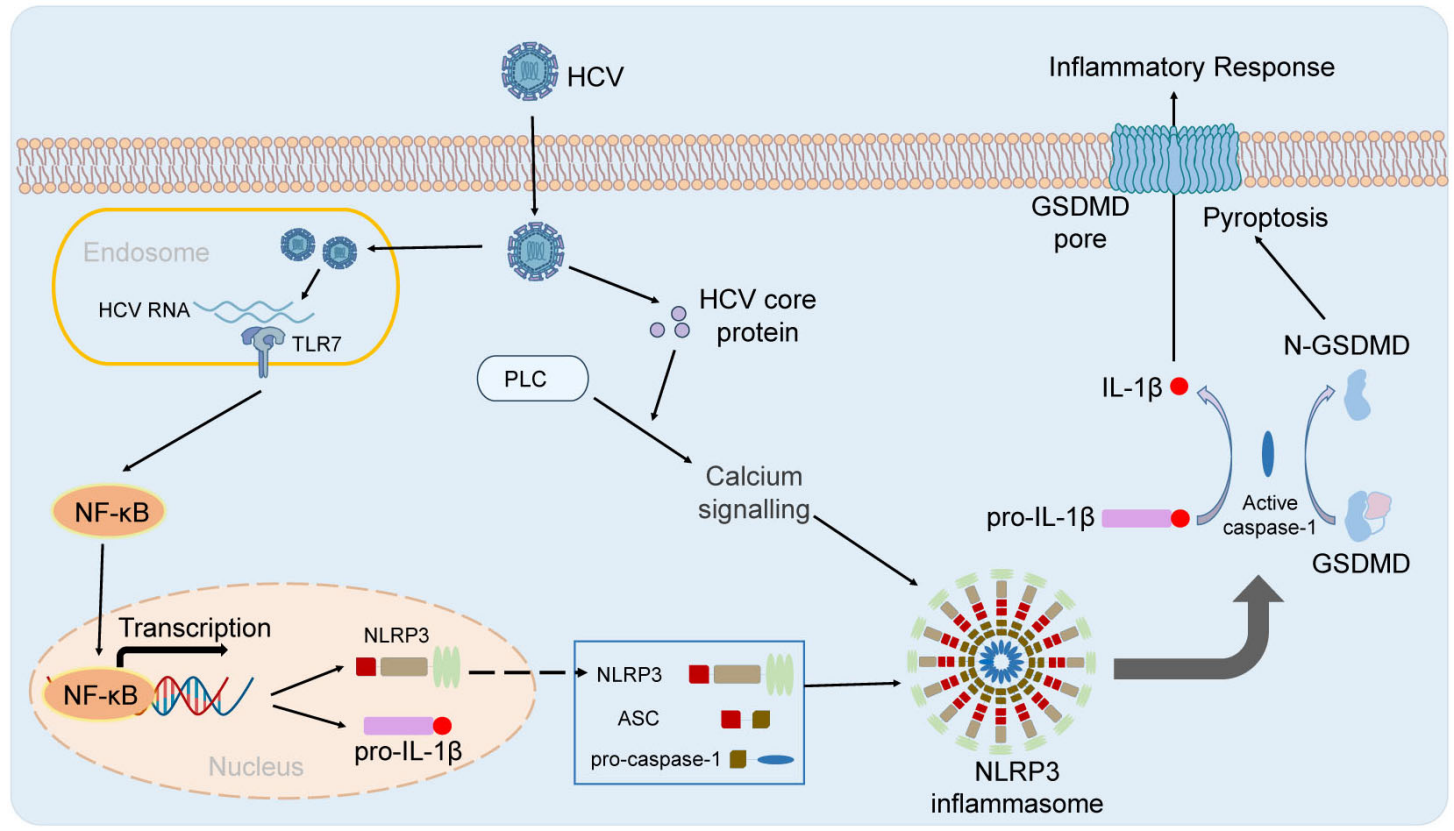
The activation of NLRP3 inflammasome in HBV infection. HCV RNA stimulates TLR7 in endosome and primes the transcription of NLRP3 genes. HCV core protein and viral infection-caused K^+^ efflux triggers the activation of NLRP3 inflammasome.

Hepatitis C virus (HCV) is one of the main causes of chronic liver disease (Axley et al., 2018). How does HCV infection activate NLRP3 inflammasome and how does NLRP3 inflammasome affect HCV infection. The Researchers suggested that HCV infection was closely involved with activation of NLRP3 inflammasome (Burdette et al., 2012; Ramachandran et al., 2021). Chen et al. showed that there was no obvious change of NLRP3 inflammasome activation in Huh7 cells and THP-1 derived macrophages infected with HCV virions (Chen et al., 2014). However, genomic RNA of HCV could activate NLRP3 inflammasome in the human myeloid cells (Chen et al., 2014). It has also been reported that NLRP3 inflammasome is activated in hepatocytes infected by HCV (Ramachandran et al., 2021). HCV infection could suppress NLRP3 inflammasome activation by deubiquitinating the NLRP3 (Ramachandran et al., 2021). Negash et al. showed that HCV could also trigger activation of NLRP3 inflammasome to initiate the hepatic inflammatory response in the Kupffer cell, and core protein of HCV could initiate activation of NLRP3 inflammasome and IL-1β release in hepatic macrophages via modulation of calcium signaling linked with phospholipase-C (PLC) activation, to drive liver inflammation (Negash et al., 2019). Glycoproteins derived from HCV, required for viral entry and fusion, could trigger NLRP3 inflammasome activation and pyroptosis in THP-1 macrophages. Seemingly, genomic RNA and some coding proteins of HCV could both activate NLRP3 inflammasome in the indicated cells. NLRP3 inflammasome also played an essential role in HCV infection and related effects. Daussy et al. suggested that HCV infection could initiate Golgi fragmentation through IRGM that mediates lipid supply for replication (Daussy et al., 2021). ASC protein at Golgi was critical for keeping IRGM under homeostasis by associating with it. HCV infection triggered dissociation of ASC from IRGM at the Golgi, and assembly of NLRP3 and ASC (Daussy et al., 2021). NLRP3 inflammasome activation also played a critical role in HCV-related liver diseases (Farag et al., 2017; Aggan et al., 2022). Aggan et al. reported that the median serum NLRP3 levels were obviously higher in HCV-infected patients compared with healthy controls, and that increased serum NLRP3 levels and hepatic expression of NLRP3 were associated with significant liver pathology. They suggested that serum NLRP3 levels could be as a potential biomarker for liver necroinflammation, fibrosis, and steatosis (Aggan et al., 2022). The p7 viroporin of HCV could contribute to liver inflammation through inducing production of IL-1β mediated by NLRP3 inflammasome activation in liver macrophages (Farag et al., 2017). Therefore, HCV regulates NLRP3 inflammasome by multiple different ways. HCV RNA stimulates TLR7 in endosome and primes the transcription of NLRP3 genes and HCV core protein and viral infection-caused K+ efflux triggers the activation of NLRP3 inflammasome (**Figure 2**). NLRP3 inflammasome play an important role in HCV infection-related diseases, which needs further specific studies.

## 4 Role of NLRP3 inflammasome activation in hepatocellular carcinoma

Chronic hepatitis B (CHB) and Chronic hepatitis C (CHC) were correlated with an high risk of cirrhosis and hepatocellular carcinoma (HCC), and CHB and CHC were likely to eventually develop into HCC (Tzeng et al., 2014; Boldeanu et al., 2020). More and more studies have suggested that activation of NLRP3 inflammasome not only participated in tumorigenesis, but also played a helpful role in some cancers (Chen et al., 2020; Zhang et al., 2020; Dai et al., 2022). Zhang et al. have indicated that Alpinumisoflavone suppresses HCC cell (SMMC 7721 and Huh7) proliferation, migration and metastasis by facilitating NLRP3 inflammasome-mediated pyroptosis (Zhang et al., 2020). Ursolic acid could inhibit the invasiveness of A498 cells by activating NLRP3 inflammasome (Chen et al., 2020). Shuanghua decoction also utilized anticancer activity by promoting activation of NLRP3 inflammasome via ROS signaling pathway in HCC cells (Dai et al., 2022). Li et al. have showed that sorcin negatively regulates pyroptosis to promote HCC proliferation, migration, and invasion by inhibiting the assembly and activation of NLRP3 inflammasome (Li et al., 2023a). However, luteoloside utilized its inhibitory effect on proliferation, invasion and metastasis of HCC cells by suppressing activation of NLRP3 inflammasome (Fan et al., 2014). Anisodamine also suppressed the growth of HCC cells by suppressing activation of NLRP3 inflammasome (Li et al., 2020). Lee et al. have showed that knockout of NLRP3 in HCC cells could suppress tumor development and metastasis, and enhance natural killer (NK) cells immunosurveillance (Lee et al., 2021). Overall, the detailed and specific mechanisms underlying the role of NLRP3 inflammasome activation in HCC development and progression needs further exploration.

## 5 The roles of other kinds of inflammasomes activation in HBV/HCV infection and related disease

The nucleotide-binding domain, leucine-rich repeat containing family caspase recruitment domain containing 4 (NLRC4), a cytosolic member of the NOD-like receptor family, is mainly activated by a range of intracellular bacteria such as *Salmonella* typhimurium and *Legionella* pneumophila (Qu et al., 2012; Jabir et al., 2015). Askari et al. reported that the expression level of NLRC4 in the peripheral blood immune cells did not change (but NLRP3 increased) in CHB patients in comparison to healthy controls, and that the NLRC4 were not significantly different among CHB patients carrying different viral loads, or between HBeAg-positive and HBeAg-negative CHB patients (Askari et al., 2016). However, we conjecture that NLRC4 inflammasomes may be activated and play a role in bacteria-co-infected Hepatitis B or Hepatitis C.

Interferon-γ-inducible protein 16 (IFI16), a member of Pyrin-Hin200 (HIN-200) family, played a critical role in immune response and antiviral activities (Almine et al., 2017). Pang et al. have suggested that IFI16 was closely related to the degree of inflammation in CHB and HBV-associated acute-on-chronic liver failure (HBV-ACLF) (Pang et al., 2018). Liu et al. have suggested that expression of IFI16 was closely related with the degree of inflammation in CHB, and the elevation of IF116 may contribute to renal damage due to their pro-inflammatory activities (Liu et al., 2022).

Absent in melanoma 2 (AIM2) is an important member of innate immune sensors that detects the presence of cytoplasmic DNA (Lugrin and Martinon, 2018). Pan et al. have suggested that the expression levels of Hepatic AIM2 were positively correlated to the severity of liver inflammation, and the expression of IL-18 was increased after AIM2 sensed HBV in hepatocytes (Pan et al., 2016). Li et al. have also showed that HBV could induce monocytes IL-18 production by activating the AIM2 inflammasome, and also induce NK cells produce IFN-γ by monocytes IL-18 (Li et al., 2023b). AIM2 also played a critical role in human hepatocellular carcinoma (HCC) (Zheng et al., 2023). Zheng et al. have suggested that the expression of AIM2 was markedly decreased in human HCC tissues compared with adjacent normal tissues, AIM2 delayed the tumor progression and correlated with immune cell infiltration (Zheng et al., 2023). Ma et al. have showed that exogenous overexpression of AIM2 in HCC cells suppressed mammalian target of rapamycin (mTOR)-S6K1 signaling pathway and further inhibited proliferation and invasion of HCC cells, and block of AIM2 in HCC cells induced mTOR-S6K1 signaling pathway activation and therefore promoted HCC progression (Ma et al., 2016). The expression of AIM2 was regulated by HBx at mRNA and protein levels, and overexpression of HBx could markedly block the expression of AIM2 at mRNA and protein levels by enhancing the stability of Enhancer of zeste homolog 2 (EZH2) (Chen et al., 2017). HBx-induced loss of AIM2 was associated with poor outcomes and promoted HCC metastasis by triggering the EMT process (Chen et al., 2017). Overall, the detailed and specific mechanisms underlying the role of AIM2 inflammasome activation in HBV/HCV infection and HCC progression needs further exploration.

## 6 Discussion

A few clinical studies have revealed the NLRP3 inflammasome play important roles in hepatitis B and C. Some studies based on *in vitro* experimental models show that NLRP3 inflammasome can be activated and regulated by HBV and HCV, and exerts both antiviral immune functions and pro-inflammatory functions. Given their roles in arousing innate immune responses, the negative regulation of inflammasome by viral components may contribute to the persistent infection. In fact, how NLRP3 inflammasome participate the pathological process of viral hepatitis, cirrhosis and HCC remains mainly unclear, which may be due to the difficulties to constructing proper research models. The studies of other kinds of inflammasomes in hepatitis B and C are also insufficient and are limited by similar difficulties. Besides, the complexity of inflammasome regulation, and multifunction of inflammasomes and their downstream effectors during HBV and HCV infections should be given enough attention. In addition, the clinical symptoms and laboratory indexes of both hepatitis B and C differ a lot at different infection stages. The study of inflammasomes should be conducted under specific pathological process of hepatitis. In a word, the research on the roles of inflammasomes in hepatitis is just beginning, and there are many technical and theoretical challenges ahead.

## Author contributions

PW: Funding acquisition, Project administration, Supervision, Writing – original draft. GY: Funding acquisition, Project administration, Supervision, Writing – original draft. QC: Writing – original draft. XZ: Writing – review & editing. YZ: Writing – original draft. ML: Writing – original draft. CL: Writing – original draft. QY: Writing – original draft. YJ: Writing – review & editing. JL: Writing – review & editing. XX: Conceptualization, Formal analysis, Funding acquisition, Project administration, Supervision, Writing – review & editing. BS: Conceptualization, Formal analysis, Funding acquisition, Project administration, Supervision, Writing – review & editing. YL: Conceptualization, Formal analysis, Funding acquisition, Project administration, Supervision, Writing – review & editing.
